# HCV Testing and Treatment of Adults in the United States: 2014 Through 2021—Data From Two National Commercial Testing Laboratories

**DOI:** 10.1111/jvh.70087

**Published:** 2025-09-29

**Authors:** Marc G. Ghany, John W. Ward, Zachary Baldwin, Shiyin Jiao, Nidhi Shukla, Arina Kuznetsova, Jatinder Kaur, Katherine J. Kosch, Timothy R. Morgan

**Affiliations:** ^1^ Clinical Hepatology Research Section, Liver Diseases Branch, National Institute of Diabetes and Digestive and Kidney Diseases, National Institute of Health Bethesda Maryland USA; ^2^ Coalition for Global Hepatitis Elimination, the Task Force for Global Health Decatur Georgia USA; ^3^ AbbVie Inc North Chicago Illinois USA; ^4^ Gastroenterology Section, VA Long Beach Healthcare System Long Beach California USA

**Keywords:** care cascade, direct acting antiviral therapies, elimination, reflex testing, screening

## Abstract

Data on the hepatitis C virus (HCV) care cascade are crucial for determining if the United States (U.S.) is on track to meet 2016 World Health Organization elimination goals. De‐identified data were analysed from persons who were screened for HCV antibody and/or tested for HCV RNA by two large U.S. commercial laboratories from 1/1/2014 to 12/31/2021. Validated imputation algorithms were used to identify persons who initiated treatment and who achieved virological cure based on viral load decline and continued negative HCV RNA test results. The 3‐digit ZIP code was used to map treatment rates by U.S. state. During 1/1/2014 to 12/31/2021, a total of 46,646,661 persons were tested for HCV antibody of whom 2,253,500 (4.8%) were positive. Among 3,117,372 persons tested for HCV RNA, 1,951,742 (62.6%) were viremic. Cumulatively, a total of 672,745/1,951,742 (34.5%) viremic persons were treated; an estimated 643,043 (96%) were cured. Treatment rates increased with older age, higher fibrosis scores, HIV positivity, residing in an urban area and in the Northeast. Persons diagnosed by reflex testing had higher treatment rates. Comparing COVID‐19 pandemic (2021) to pre‐pandemic (2019) periods, 24% more HCV antibody tests were performed (10,167,524 vs. 7,727,318), but fewer persons were treated (21,136 vs. 26,124, 23% decline) and cured (19,584 vs. 24,480, 25.0% decline) in 2021, respectively. In 2021, primary care providers diagnosed and treated the greatest proportion of persons. Treatment uptake across the U.S. remains low, underscoring the need for additional measures to expand access to testing and treatment, necessary to reach the U.S. goals for HCV elimination by 2030.

**Abbreviations**
CDC: Centers for Disease Control and Prevention
DAAs: direct acting antivirals
HCC: hepatocellular carcinoma
HCV: hepatitis C virus
HIV: human immunodeficiency virus
U.S: United States
USPSTF: U.S. Preventive Services Task Force
WHO: World Health Organization

## 1 Introduction

Chronic hepatitis C virus (HCV) infection is a major cause of premature mortality from cirrhosis, hepatocellular cancer (HCC) and extra‐hepatic co‐morbidities. Globally, an estimated 50 million persons have chronic infection, resulting in 242,000 deaths annually [1]. The United States (U.S.) has a large burden of HCV infection, ranked fifth highest in prevalence globally and second highest among high‐income countries [2]. In 2020, an estimated 2.4–4 million persons in the U.S. were infected with HCV, causing 12,717 deaths from HCV‐related liver disease [3]. All oral direct‐acting antiviral therapies (DAAs) that can cure more than 95% of persons with chronic infection were approved in October 2014. Consequently, all persons with HCV infection, except those with a short life expectancy, are now recommended to receive antiviral treatment.

The high burden of HCV infection and related mortality coupled with the availability of curative therapies prompted the World Health Organization (WHO) to set goals for the elimination of hepatitis C with implementation of prevention and care services to diagnose ≥ 90% and treat ≥ 80% of HCV infected persons [4]. In the U.S., the Viral Hepatitis National Strategic Plan: A Roadmap to Elimination 2021–2025 sets targets for reducing HCV transmission, prevalence and mortality [5]. To reach goals for HCV elimination, there needs to be a large scale‐up in HCV screening, diagnosis, linkage to care and treatment. To achieve this, from 2012 to 2020, the Centers for Disease Control and Prevention (CDC) and the U.S. Preventive Services Task Force (USPSTF) recommended one‐time HCV testing for all persons born between 1945 and 1965 (birth cohort screening) because of the higher prevalence of chronic HCV infection in this age group compared to other birth cohorts. In 2020, in response to rising HCV incidence, driven predominantly by increases in injection drug use, the CDC and USPSTF recommended HCV testing for all persons 18–79 years of age [6, 7].

To track the effectiveness of these policy changes, data are needed to monitor the number of persons tested, diagnosed and treated for hepatitis C infection. However, because of limited capacity, few state and local public health systems collect these data. To meet this need, health officials have formed public‐private partnerships with commercial laboratories to collect and analyse de‐identified data from laboratory‐based testing for HCV. To assist the use of commercial laboratory data for HCV surveillance, the CDC developed an algorithm to monitor trends in screening for HCV antibody; HCV PCR testing; detection of HCV viremia and evidence of virologic cure. Using this algorithm, a CDC study of data collected from 2014 to 2021 by one large commercial laboratory revealed a substantial loss of patients moving across the HCV care cascade resulting in only approximately one third of persons diagnosed with an HCV infection receiving curative therapy [8].

Two additional studies have examined data compiled from the records of two large commercial laboratories including the records from the commercial laboratory used in the CDC analysis [9, 10]. In the earliest study, data from persons tested from 2013 to 2016 found increases in numbers of persons tested for HCV, and who were viremic. Over the study period, the proportion of viremic persons treated increased from 6.6% in 2013 to 22.3% in 2016 [9]. A second study of persons tested in 2017 to 2019 found increasing numbers of viremic patients who were < 40 years with mild liver disease and 26.8% of viremic persons were treated [10]. Of note due to the lack of pharmacy data, both studies relied on use of a validated algorithm to identify treatment‐related HCV cure.

Data from the two commercial laboratories are now available for the years 2020 and 2021. We report the findings of an evaluation of the data set for the 2014–2021 HCV care cascade to explore trends in testing and treatment, including the type of clinical providers ordering the HCV PCR testing, and the impact of reflex HCV RNA testing on the diagnosis of HCV infection and receipt of HCV therapy. The data also provide the opportunity to assess the impact of the response to the COVID‐19 pandemic on HCV testing.

## 2 Methods

### 2.1 Data Source and Patient Population

This study used a secondary, de‐identified dataset combined from two large U.S. laboratory companies (LabCorp and Quest Diagnostics) that provide HCV testing across all 51 U.S. states and territories (Puerto Rico and District of Columbia (D.C.)). This derived dataset represents the largest available HCV laboratory dataset in the U.S. Records of all adults who were screened for HCV antibody and/or tested for HCV RNA from 1/1/2014 to 12/31/2021 were included in this analysis. Not all individuals had both HCV antibody and HCV RNA testing. HCV testing could be ordered as HCV antibody alone, HCV RNA alone, or reflex testing (i.e., if HCV antibody is detectable then HCV RNA is tested on the same blood sample). For this analysis, data to assess the selection of reflex testing was only available from one laboratory, LabCorp.

Patient characteristics, including age (in years), sex (female, male, or unknown) and region of residence (Northeast, Southeast, Midwest, Southwest and West) were available for all individuals included in the analysis. To map HCV treatment rates by the U.S. state, an individual's region of residence was determined by the location of HCV RNA testing and detection of viremia (diagnosis) using the 3‐digit ZIP code. For individuals who tested positive for HCV RNA, additional information was retrieved from both laboratory datasets for the following variables: HCV genotype; urbanisation (rural or urban); human immunodeficiency virus (HIV) diagnosis; laboratory results for calculation of the FIB‐4 score (alanine aminotransferase, aspartate aminotransferase and platelets). To assess fibrosis stage, FIB‐4 scores were grouped as: minimal or none < 1.45, moderate 1.45 to 3.25, advanced > 3.25 [11]. Clinical care providers ordering the HCV RNA testing were grouped as HCV specialists (gastroenterologists, infectious disease specialists, hepatologists), other clinical specialties (addiction medicine, psychiatry, behavioural health, emergency medicine, prison medicine, pain management, mental health, community health, rural health), primary care physicians, nurse practitioners, physician assistants and all other providers.

### 2.2 Algorithms for Treatment Receipt and Achievement of Sustained Virologic Response

#### 2.2.1 Treatment Receipt

Due to the lack of information on the treatment of hepatitis C and continuity of medical or pharmacy benefit enrollment in the derived dataset, receipt of HCV treatment and achieving sustained virologic response (SVR) (i.e., cure) was imputed using validated algorithms based on viral load decline and continued negative HCV RNA test results, respectively [9]. A viral load decline of at least 1.2 × log_10_ units (minimum meaningful decline attributable to treatment) [9] was used to identify patients assumed to have initiated therapy. Briefly, machine learning predictive models were built and validated using a separate set of 92,099 treated HCV individuals with medical and pharmacy claims available in the Symphony Health Solutions (SHS) medical and pharmacy claims dataset from 2017 to 2019, with over 98% accuracy in each year [9]. This model—now validated across 7 years consecutively with > 98% accuracy—was then applied to our dataset; individuals in the current laboratory dataset who were predicted to have achieved SVR from the machine learning algorithms were classified as cured in the year following the year of treatment (e.g., individuals flagged as initiating treatment in 2019 were classified as treated in 2019 and cured in 2020).

#### 2.2.2 Viremic Status

Because individuals might have varied follow up duration and/or inconsistent HCV RNA measurements in the years from 2014 to 2021, a longitudinal method was applied to impute their HCV viremic status over multiple years. Individuals who had two positive HCV RNA values with gap years in between were assumed to stay HCV viremic in the gap years.

### 2.3 Observed Rates of HCV Screening and Diagnostic Testing

The number of persons screened for HCV antibody and the number who tested positive for HCV antibody or for HCV RNA were assessed for each year from 2014 to 2021. The proportion of individuals who were HCV antibody positive among all individuals tested for HCV antibody was calculated.

### 2.4 HCV Antibody With Reflex to HCV RNA Testing

Ordering of reflex testing (HCV antibody with reflex to HCV RNA) was identified using test numbers, test names, and by matching the test date (date the specimen was drawn) of the HCV antibody test with the date of the HCV RNA test.

### 2.5 Statistical Analysis

This study was descriptive in nature. After retrieving and combining data from the two laboratory databases, observations were reported for the number of individuals in whom HCV antibody was tested, who were HCV antibody positive, persons tested for HCV RNA, HCV RNA positive, as well as those treated and cured individuals across the years 2014 to 2021 (henceforth termed the “HCV Care Cascade”). The HCV care cascade during this period was also reported based on sex and fibrosis stage categorisations. HCV cured individuals predicted from the machine learning algorithms were removed from each year's estimates. The total number of persons who remained HCV RNA positive and not cured was reported for the respective years between 2014 and 2021. To assess the impact of COVID‐19, changes in HCV treatment and cure rates between 2019 and 2021 were reported for HCV RNA tested, HCV RNA positive, treated (among those who tested HCV RNA+ in 2021), and cured individuals who had an HCV Ab+ test in 2021. The observed number of individuals who were HCV antibody screened, tested HCV antibody positive and tested HCV RNA positive was stratified by age for the year 2021. The proportion of RNA positive patients who were treated in 2021 were reported as a geographic heatmap of the 51 U.S. states and territories (Puerto Rico and D.C.). Observations of provider specialties that ordered the PCR test detecting HCV viremia and treated overall are reported for 2021. Ordering provider of the first positive RNA test in 2021 was assumed to be the diagnosing provider. Ordering provider of the RNA test with a decline of at least 1.2 × log_10_ units since the first positive HCV RNA test was assumed to be the treating provider. Observations are reported on an annual basis from 2014 to 2021. Time to treatment after a reflex test was analysed and was limited to individuals with an HCV antibody positive test at least 28 days prior to the viral load decline.

Means and standard deviations were reported for normally distributed continuous variables, medians and interquartile ranges for non‐normally distributed continuous variables, and frequencies and percentages (%) for categorical variables. All persons identified as RNA+ were included in the analysis. For demographical and clinical characteristics, persons with missing data in any field were not removed, but rather they were not counted toward the denominator and did not contribute to the percentage calculation for that characteristic.

### 2.6 Software

Data cleaning and manipulation were performed using SAS 9.4 (Cary, NC, USA). Machine learning algorithms were developed in R software (R Foundation for Statistical Computing, Vienna, Austria). Compliance with Ethics Guidelines.

### 2.7 Compliance With Ethics Guidelines

With permission from Symphony Health, an ICON plc Company, PatientSource (for LabCorp data) and Quest Diagnostics, this study utilised de‐identified retrospective data from two U.S. laboratory datasets. Because the data were de‐identified, no ethics committee approval was required. Additionally, as this study is based on laboratory data, it does not contain any new studies with human or animal subjects performed by any of the authors.

## 3 Results

**FIGURE 1:**
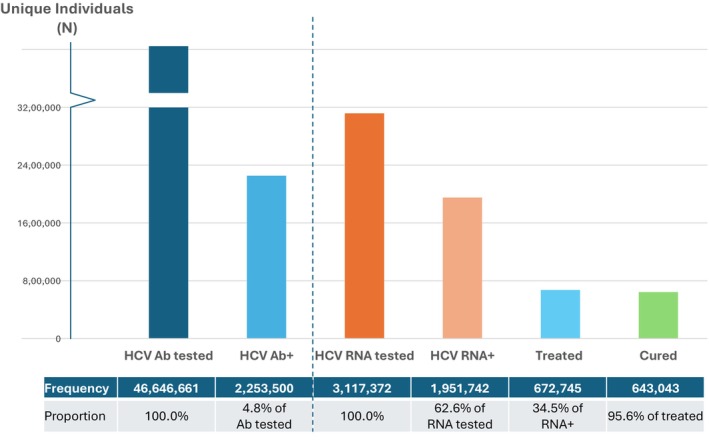
HCV care cascade, 2014–2021 cumulative. HCV treated and cured individuals were identified using validated imputation algorithms. HCV, hepatitis C virus.

**FIGURE 2:**
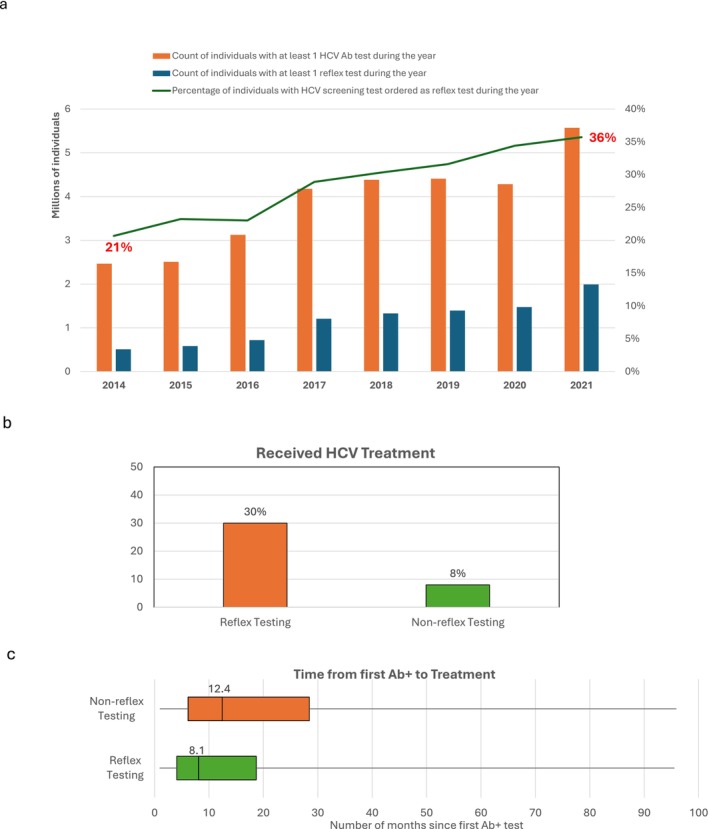
(a) Ordering of HCV Ab with reflex to HCV RNA testing among HCV tested adults in the US, 2014–2021. Ordering of reflex testing (HCV Antibody with reflex to RNA test) was identified using test numbers, test names, and by matching the test date (date the specimen was drawn) of the Antibody test with that of the RNA test. Reflex testing analyses are only available with data from one large US national laboratory. (b) Association of Reflex Testing, 2014–2021. Reflex testing (HCV Antibody with reflex to RNA test) was identified by matching the test date (date the specimen was drawn) of the Antibody test with that of the RNA test. Reflex testing analyses are only available with data from one large US national laboratory. Percent treated for individuals for whom Ab and RNA testing were ordered separately may be underestimated due to inclusion of those who may not have a confirmed RNA+ test result. (c) Receipt of HCV Treatment, 2014–2021. Receipt of treatment was determined based on a viral load decline of at least 1.2 × log_10_ units since the first positive HCV RNA test, indicating that treatment was initiated in the immediate period prior to the decline. Time to treatment analysis was limited to individuals with an Ab+ test at least 28 days prior to the viral load decline.

During the period January 1, 2014, through December 31, 2021, a total of 46,646,661 individuals were tested for hepatitis C antibody at the two nationwide commercial laboratories. Of the individuals tested, 2,253,500 (4.8%) were HCV antibody positive. During the same period, 3,117,372 persons were tested for HCV RNA and 1,951,742 (62.6%) were viremic (Figure 1). The number of individuals tested for HCV antibody increased each year from 2014 through 2019 (Figure 2). The number of HCV antibody tests decreased slightly in 2020, likely reflective of the impact of the COVID‐19 pandemic followed by an increase in 2021. From 2014 through 2021, the proportion of individuals who received reflex HCV RNA testing of HCV Ab+ specimens rose from 21% in 2014 to 36% in 2021 (Figure 2). A greater proportion of men tested positive for HCV RNA compared to women, 63.1% versus 36.8%, respectively (Table 1).

**TABLE 1:** Baseline characteristics of HCV RNA‐positive adults, 2014–2021.

Variable	Total (*N* = 1,744,908)^a^ [including unknown/missing gender (0.1%)]	Male [*N* = 1,100,928 (63.1%)]	Female [*N* = 642,155 (36.8%)]
Mean age^b^ (Years) [(±SD)]	48.9 (±14.2)	49.5 (±13.8)	47.8 (±15.0)
18–30	242,330 (13.9)	132,502 (12.0)	109,574 (17.1)
31–50	584,564 (33.5)	367,820 (33.4)	216,166 (33.7)
51–64	696,920 (39.9)	460,896 (41.9)	235,291 (36.6)
≥ 65	221,094 (12.7)	139,710 (12.7)	81,124 (12.6)
HCV genotype, *n* (%)^c^			
1	951,512 (73.2)	606,618 (73.8)	343,957 (72.4)
2	144,620 (11.1)	88,680 (10.8)	55,794 (11.7)
3	181,229 (14.0)	113,723 (13.8)	67,317 (14.2)
4	14,086 (1.1)	8774 (1.1)	5298 (1.1)
5	169 (0.0)	87 (0.0)	81 (0.0)
6	5106 (0.4)	3009 (0.4)	2091 (0.4)
Mixed	1963 (0.2)	1274 (0.2)	689 (0.1)
Fibrosis stage (FIB‐4 score), *n* (%)^c^			
No/Minimal (FIB‐4 score < 1.45)	780,093 (51.8)	470,754 (49.6)	308,562 (55.7)
Moderate (1.45–3.25)	327,869 (21.8)	210,739 (22.2)	116,776 (21.1)
Advanced (FIB‐4 score > 3.25)	397,839 (26.4)	268,529 (28.3)	128,950 (23.3)
HIV status, *n* (%)^c^			
Negative	1,706,305 (97.8)	1,073,244 (97.5)	631,266 (98.3)
Positive	38,603 (2.2)	27,684 (2.5)	10,889 (1.7)
Region of U.S.,^d^ *n* (%)^c^ (based on 3‐digit zip code)			
Northeast	339,298 (20.6)	213,470 (20.6)	125,499 (20.5)
Southeast	548,451 (33.2)	320,469 (30.9)	227,763 (37.1)
Midwest	238,593 (14.5)	146,136 (14.1)	92,353 (15.1)
Southwest	169,730 (10.3)	109,488 (10.6)	60,133 (9.8)
West	354,953 (21.5)	246,994 (23.8)	107,532 (17.5)
HCV RNA+ test ordering provider specialty^e^, *n* (%)^c^			
HCV specialists (gastroenterologists, infectious disease specialists, hepatologists)	251,651 (25.0)	150,108 (23.9)	101,352 (27.0)
Emerging HCV specialties (addiction medicine, psychiatry, behavioural health, emergency medicine, prison medicine, pain management, mental health, community health, rural health)	35,320 (3.5)	22,821 (3.6)	12,403 (3.3)
Primary care physicians (family medicine, internal medicine, hospitalists, pharmacists, general surgery, paediatrics, obstetrician/gynaecologist, geriatrics, Nurse Practitioner, Physician Assistant)	647,963 (64.5)	412,340 (65.6)	235,141 (62.4)
Any other	70,044 (7.0)	44,097 (7.0)	25,860 (6.9)
Urban–rural status^f^, *n* (%)^c^			
Urban (1 = Large central metro, 2 = Large fringe metro, 3 = Medium metro, 4 = Small metro)	1,163,166 (76.2)	736,538 (76.9)	425,665 (74.9)
Rural (5 = Micropolitan, 6 = non‐core)	364,119 (23.8)	221,244 (23.1)	142,705 (25.1)

^a^ Total of HCV RNA positive individuals includes only adults.

^b^ Age for each patient is defined as age during the year of their first positive HCV RNA test.

^c^ Percentages are calculated using non‐missing observations as the denominator.

^d^ Northeast: Maine, Massachusetts, Rhode Island, Connecticut, New Hampshire, Vermont, New York, Pennsylvania, New Jersey, Delaware, Maryland. Southeast: West Virginia, Virginia, Kentucky, Tennessee, North Carolina, South Carolina, Georgia, Alabama, Mississippi, Arkansas, Louisiana, Florida. Midwest: Ohio, Indiana, Michigan, Illinois, Missouri, Wisconsin, Minnesota, Iowa, Kansas, Nebraska, South Dakota, North Dakota. Southwest: Texas, Oklahoma, New Mexico, Arizona. West: Colorado, Wyoming, Montana, Idaho, Washington, Oregon, Utah, Nevada, California, Alaska, Hawaii.

^e^ HCV Specialists includes gastroenterologists, infectious disease specialists and hepatologists. Emerging Specialties includes addiction medicine, psychiatry, behavioural health, emergency medicine, prison medicine, pain management, mental health, community health and rural health. Primary Care Physicians includes family medicine, internal medicine, hospitalists, pharmacists, general surgery, paediatrics, obstetrician/gynaecologist and geriatrics.

^f^ Urban/rural status of patient residence was defined using the 2013 NCHS 6‐Level Urban–Rural Classification Scheme (ranging from most urban to most rural: large central metropolitan, large fringe metropolitan, medium metropolitan, small metropolitan, micropolitan and noncore). Reference: Ingram and Franco [12]. https://www.cdc.gov/nchs/data_access/urban_rural.htm.

Cumulatively, from 2014 through 2021, a total of 672,745 individuals were treated for HCV infection. An estimated 643,043 (96%) were cured of their HCV infection (Figure 1). The treatment rate of HCV RNA + persons did not vary by gender or by genotype. However, HCV treatment rates increased stepwise by age from 21% among individuals 18–30 years to 56% among individuals > 65 years (Table S1 and Figure S1a). HCV treatment rates also varied by the fibrosis stage from 46.5% among persons with FIB‐4 scores > 3.25 (indicative of advanced liver fibrosis) compared to 33.6% among individuals with FIB‐4 scores < 1.45 (indicative of no/mild fibrosis), (Table S1 and Figure S1b). A greater percentage of HIV‐infected individuals received HCV treatment (62.5%) as compared with HIV‐uninfected individuals (38%) (Table S1 and Figure S1c). The percentage of individuals treated varied by U.S. region, with the highest percentage in the Northeast (47.3%) and the lowest percentage in the Midwest (27.5%) (Table S1). State‐by‐state differences in treatment rates also occur, (Figure S2). A greater proportion of individuals in urban areas received treatment (40.1%) as compared with rural areas (35.4%) (Table 1). HCV specialists treated the highest percentage of HCV RNA positive individuals in their care (76.2%) followed by primary care providers (31.4%) and nurse practitioners/physician assistants (27%). Compared to individuals for whom HCV antibody and HCV RNA testing were ordered separately, those identified as HCV RNA + by reflex testing of the HCV antibody positive specimen were more likely to receive HCV treatment (30% vs. 8%) (Figure 2) and to receive treatment sooner 8.1 vs. 12.4 months, respectively (Figure 2).

**FIGURE 3:**
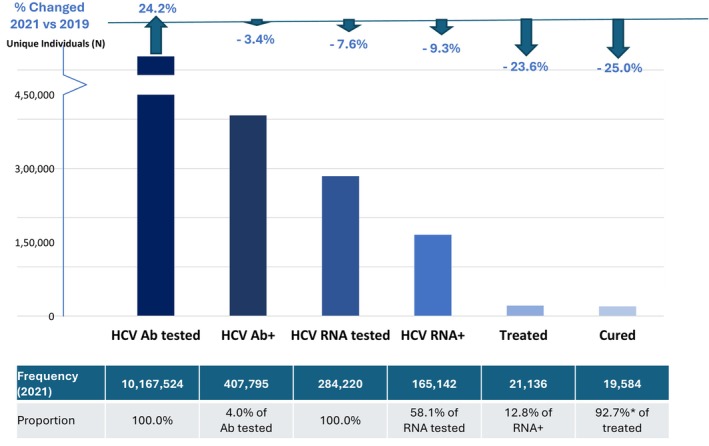
Changes in HCV treatment and cure, 2021 vs. 2019. Figure is only showing HCV RNA tested, RNA+, treated (among those tested RNA+ in 2021), and cured individuals who had an Ab+ test in 2021. HCV treated and cured individuals were identified using validated imputation algorithms. *Cure rate may be underestimated because patients diagnosed toward the end of 2021 may not have enough follow‐up data to predict treatment initiation and cure. HCV, hepatitis C virus.

**FIGURE 4:**
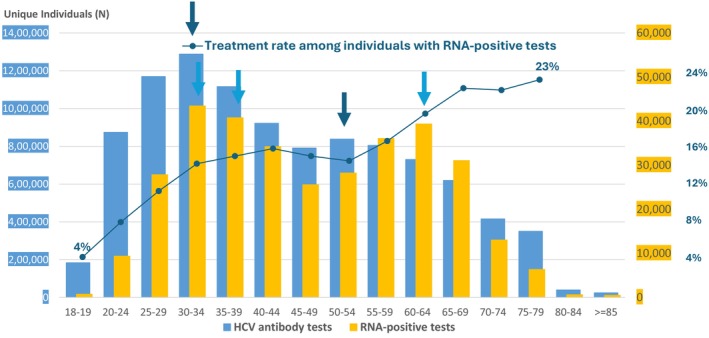
Age distribution for individuals with HCV antibody screening and RNA‐positive tests among adults in the US in 2021. Receipt of treatment was determined based on a viral load decline of at least 1.2 × log_10_ units since the first positive HCV RNA test, indicating that treatment was initiated in the immediate period prior to the decline.

In 2021, the most recent year of observation, a total of 10,167,524 persons were tested for HCV antibody, of whom 407,795 (4%) were positive; 284,220 persons were tested for HCV RNA, of whom 165,142 (58.1%) were positive. An estimated 21,136 (12.8%) HCV RNA positive persons were treated, of whom 19,584 (92.7%) were cured (Figure 3). In 2021, the number of persons screened for HCV antibody exhibited a bimodal age distribution, with age group 30–34 years representing the largest number (1,287,974) tested, and another peak in the age group 50–54 years (838,009). The age distribution of persons who tested positive for HCV RNA displayed a similar bimodal pattern, with the highest positivity rate among those aged 30–39 years followed by those aged 60–64 years (Figure 4).

**FIGURE 5:**
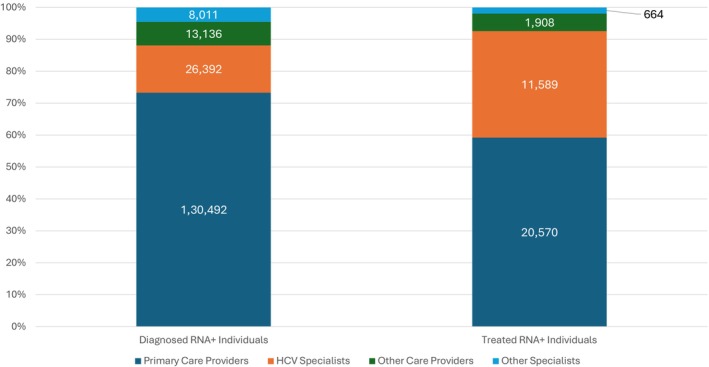
Percent of RNA+ patients diagnosed/treated by specialty in 2021. Primary Care Physicians includes family medicine, internal medicine, hospitalists, pharmacists, general surgery, paediatrics, obstetrician/gynaecologist and geriatrics. HCV Specialists includes gastroenterologists, infectious disease specialists and hepatologists. Emerging Specialties includes addiction medicine, psychiatry, behavioural health, emergency medicine, prison medicine, pain management, mental health, community health, rural health.

To look for trends in the care cascade related to the COVID‐19 pandemic, testing and treatment rates from 2021 (pandemic) were compared to results in 2019 (pre‐pandemic). In 2021, a total of 24% more HCV antibody tests were performed as compared with 2019 (Figure 3). However, in 2021, there was a 7.6% decrease in the proportion of persons tested for HCV RNA. Compared to 2019, the proportion of persons treated and cured in 2021 declined by 23.6% and 25.0%, respectively, although cure rates remained high (92.7%) (Figure 3). In 2021, primary care providers diagnosed the greatest number of persons with HCV viremia (73.3% of all HCV RNA positive tests), followed by HCV specialists (14.8%) and other clinical specialties care providers 7.4% (Figure 5). In 2021, primary care providers also treated the greatest proportion of persons 59.2%, followed by HCV specialists 33.4% with a minority of persons treated by other providers/specialists 7.4% (Figure 5).

## 4 Discussion

An analysis of the HCV care cascade was conducted using recent available data from two commercial laboratories to assess progress of HCV elimination in the U.S. Alarmingly, only 35% of HCV RNA + individuals received treatment. More positively, the analysis revealed 96% of persons treated were cured of their HCV infection.

The data from this analysis are consistent with results of a CDC analysis of the HCV care cascade, using data from a single national laboratory (Quest Diagnostics), reporting that only 33% of individuals with chronic HCV infection have been cured [8]. The analysis highlights that substantial interruptions in the HCV care cascade continue to exist, resulting in low treatment uptake across the U.S. These interruptions are likely due to the persistence of provider, patient and health system barriers to initiation of therapy. The greatest loss of persons appears to be at the stage of linkage to care and initiation of treatment. For the U.S. to achieve the national goal of reducing HCV cases by 80% by 2030, additional measures are needed to expand access to treatment [13].

Similar to previous results, the greatest number of positive HCV antibody tests was found in two age groups, 30–39 years and 60–64 years. The peak among younger individuals likely represents infections associated with the emergence of the opioid epidemic and increase in injection drug use and supports the CDC 2020 recommendations to expand HCV screening to all adults 18 and older. The second peak corresponds to CDC‐defined risk for people born 1945 through 1965 (“birth cohort”) with greater risk for nosocomial transmission prior to HCV viral discovery and implementation of blood bank screening and other prevention measures. However, while screening increased in 2021 in our cohort, there was a 25% decrease in the number of persons being treated and cured suggesting continued limitations in access to HCV treatment, possibly an adverse consequence of the COVID‐19 pandemic.

Although treatment rates were higher among persons with advanced fibrosis and HIV, likely reflecting the greater urgency for treatment in these populations, < 50% of persons with advanced fibrosis/cirrhosis received treatment. This is a subgroup that should be targeted for urgent therapy due to the increased risk for progression to decompensation or HCC. Notably, reflex testing for HCV RNA of HCV antibody positive specimens was associated with higher rates of linkage to care and shortened duration of time from detection of HCV viremia to treatment. Additionally, in 2021, there appeared to be a shift in health providers who were performing testing and prescribing treatment, from specialists to primary care providers, with the latter treating almost 60% of persons with active infection. This shift in healthcare providers from specialists to primary care may alleviate the shortage of providers. However, the marked geographical differences in the rate of treatment uptake across the U.S. with a noted urban–rural divide, findings similar to the CDC analysis, suggest that health system barriers remain [13]. States that have removed restrictions to treatment, such as requirements for sobriety or presence of fibrosis and have employed novel models of paying for treatment, such as a subscription‐based model (“Netflix” model) [14], have higher rates of treatment compared to states that continue to impose restrictions on access to treatment. Interestingly, the treatment rate was linearly related to age, being lowest among 18–30 years and highest among those > 65 years. This finding is similar to that reported from an insured population where treatment initiation was lowest among adults aged 18–29 and 30–39 years with Medicaid or private insurance, compared with those aged 50–59 years after adjustment for insurance type [15]. This suggests that younger persons may be less inclined to prioritise treatment or might experience greater difficulties in accessing treatment compared to older persons.

The commercial laboratory data contrast sharply with that from the Veteran's Health Administration (VHA) which has implemented strategies to successfully test more than 80% of the 1945–1965 birth cohort, and treat > 80% of HCV RNA positive patients with a documented cure rate > 90% [16]. The VHA accomplished this by negotiating drug prices and having funding to cover the cost of treatment, removing treatment barriers such as the need for prior authorisations, patient copays and removing treatment restrictions such as active substance abuse or alcohol sobriety. Additionally, in 2009, the VHA implemented reflex HCV RNA PCR testing, and greatly expanded and educated the number of clinical pharmacists and other clinicians who could prescribe and manage the care of persons with HCV infection. The VA also developed robust systems for tracking patients entering the Care Cascade. The success of the VHA model serves as a model for other health systems in the U.S. to emulate. The U.S. can also learn from other national programs. Egypt, a middle‐income country, and Rwanda are examples of countries that have integrated public health and clinical care approaches. In Egypt this has yielded a dramatic reduction in the prevalence of hepatitis C through its “100 million Healthy Lives” nationwide campaign [17].

To counter the low rate of HCV treatment nationally, key U.S. stakeholders proposed a National HCV Elimination Plan in 2023–2024 [18]. The elimination plan has four key elements: (1) to shorten time to diagnosis through use of point‐of‐care testing that provides rapid results, (2) to remove burdensome criteria to qualify for treatment, (3) to lower drug costs through the use of subscription models and finally (4) to invest in community health programmes to deliver care and to develop a vaccine. Congressional action is needed to implement the plan.

Obtaining data to derive the care cascade remains a challenge. States are required to report HCV cases through the National Notifiable Disease Surveillance System. However, many states underreport HCV cases due to limited resources, asymptomatic infections, lack of access to healthcare and limitations in surveillance systems. Our analysis suggests that despite recognised limitations, data from commercial laboratories can provide useful information on the care cascade to inform progress on HCV elimination.

Our analysis has several strengths. There was large coverage of the U.S. population, including data collected from all 51 states and territories, through two datasets providing robustness and generalisability of the findings. The ability to evaluate trends between pre‐pandemic and pandemic periods identified areas for improvement for meeting WHO targets. However, the two commercial laboratories do not represent all persons tested for HCV over the observation period. Data on drug prescriptions are lacking and results on individual treatment initiation and “cure” had to be inferred using an algorithm relying on decline of HCV RNA. However, the validity of this algorithm has been confirmed [13]. Although persons with acute HCV infection can mount an immunologic response that results in viral clearance, the proportion of persons with acute HCV infection is typically a small proportion of patients with HCV infection. The proportion of persons treated for whom HCV antibody and RNA testing were ordered separately may be underestimated due to inclusion of those who may not have a confirmed HCV RNA positive test result. Similarly, cure rates for 2021 may have been underestimated because data on HCV RNA results from individuals who began DAA treatment in late 2021 needed to determine cure, were unavailable. HCV RNA data obtained after assessment of cure were incomplete; therefore, we could not provide data on reinfection rates. Finally, we did not have complete data on insurance status of the cohort and therefore could not analyse how this affected the care cascade.

In conclusion, data based on two large commercial laboratories indicate that only slightly more than one third of individuals with diagnosed HCV infection were treated for their HCV infection from 2014 to 2021. For the U.S. to reach goals for HCV elimination, linkage to treatment for persons diagnosed with HCV infection must improve. Our analysis suggests that several simple measures may provide large dividends in the U.S. elimination efforts. These include expanding, through training, the number of primary care providers treating HCV infection, removal of restrictions to accessing treatment [19], implementation of reflex HCV RNA testing [20], and updating the diagnostic algorithm to include the use of rapid point‐of‐care tests, which may reduce loss of persons between diagnosis and linkage to care. The U.S. has all the tools required for HCV elimination—now is the time to act.

## Author Contributions

M.G.G., J.W.W. and T.R.M.: study concept and design; analysis and interpretation of data; drafting of the manuscript; critical revision for intellectual content. Z.B.: analysis and interpretation of data; statistical analysis; critical revision for intellectual content. S.J. and A.K.: acquisition of data; analysis and interpretation of data; statistical analysis; drafting of the manuscript; critical revision for intellectual content. J.K.: study concept and design; analysis and interpretation of data; critical revision for intellectual content. K.J.K.: drafting of the manuscript; critical revision for intellectual content.

## Conflicts of Interest

Marc G. Ghany: None. John W. Ward: The Task Force for Global Health receives funds for the general support of the Coalition for Global Hepatitis Elimination from Abbott, Gilead, AbbVie, Dynavax, Merck, GSK, Siemens, Cepheid, Roche, Pharco, Zydus Life Sciences, governmental agencies and philanthropic organizations. Dr. Ward received no compensation for authorship of this manuscript. Nidhi Shukla: employed by AbbVie. Arina Kuznetsova, Zachary Baldwin, Shiyin Jiao, Jatinder Kaur and Katherine Kosch: employees of AbbVie and may own stock. Timothy R. Morgan: none.

## Supporting information


**Table S1:** Rates of treatment initiation among RNA‐positive adult patients by baseline characteristics, 2014–2021.

**Figure S1:** (a) HCV treatment rates among HCV RNA‐positive patients by age, 2014–2021. (b) HCV treatment rates by FIB‐4 score, 2014–2021. (c) HIV co‐infection status, 2014–2021.
**Figure S2:** Percentage of the HCV RNA‐positive patients treated in 2021 receipt of treatment was determined based on a viral load decline of at least 1.2 × log_10_ units since the first positive HCV RNA test, indicating that treatment was initiated in the immediate period prior to the decline.

## Data Availability

The data that support the findings of this study are available on request from the corresponding author. The data are not publicly available due to privacy or ethical restrictions.
