# Supplementary material for: HCV Testing and Treatment of Adults in the United States: 2014 Through 2021—Data From Two National Commercial Testing Laboratories
Source: J Viral Hepat. 2025 Sep 29;32(11):e70087. doi: 10.1111/jvh.70087 (PMC12477661; doi:10.1111/jvh.70087)
Supplement: Supplementary file 1 — Table S1: Rates of treatment initiation among RNA‐positive adult patients by baseline characteristics, 2014–2021. [file JVH-32-0-s002.docx]

Supplementary Table 1: Rates of Treatment Initiation among RNA-positive Adult Patients by Baseline Characteristics, 2014-2021*

| **Variable** | **Number of patients who initiated treatment at any time after first HCV RNA+ test**  (Total N = 672,745) | **Rate of Treatment Initiation**  (Number of patients who initiated treatment divided by number of patients who had a positive RNA test  (672,745 treated out of 1,744,908 RNA-positive patients = 38.5%) |
| --- | --- | --- |
| Mean Age (Years) [(±SD) (Range)] | 53.3 (±13.0) (18-107) |  |
| 18-30 | 51,339 | 21.2% |
| 31-50 | 172,691 | 29.5% |
| 51-64 | 324,251 | 46.5% |
| ≥65 | 124,464 | 56.3% |
|  |  |  |
| Gender |  |  |
| Male | 419,966 | 38.1% |
| Female | 250,852 | 39.1% |
|  |  |  |
| HCV genotype |  |  |
| 1 | 2,342 | 33.2% |
| 1A | 301,175 | 38.6% |
| 1B | 74,577 | 45.6% |
| 2 | 56,189 | 38.9% |
| 3 | 61,010 | 33.7% |
| 4 | 5,809 | 41.2% |
| 5 | 74 | 43.8% |
| 6 | 2,755 | 54.0% |
| Mixed | 719 | 36.6% |
|  |  |  |
| Fibrosis stage  (FIB-4 score), n (%) |  |  |
| No/Minimal (FIB-4 score < 1.45) | 262,272 | 33.6% |
| Moderate (1.45-3.25) | 140,392 | 42.8% |
| Advanced (FIB-4 score > 3.25) | 184,944 | 46.5% |
|  |  |  |
| HIV status |  |  |
| Negative | 648,562 | 38.0% |
| Positive | 24,111 | 62.5% |
|  |  |  |
| Region of U.S. (Based on state) |  |  |
| Northeast | 160,652 | 47.3% |
| Southeast | 216,542 | 39.5% |
| Midwest | 65,581 | 27.5% |
| Southwest | 64,787 | 38.2% |
| West | 149,201 | 42.0% |
|  |  |  |
| HCV RNA+ Test Ordering Provider Specialty |  |  |
| HCV Specialists | 191,761 | 76.2% |
| Emerging Specialties | 5,019 | 14.2% |
| Primary Care Physicians | 145,361 | 31.4% |
| Nurse Practitioner | 36,604 | 26.5% |
| Physician Assistant | 14,264 | 30.3% |
| Other | 21,835 | 31.2% |
|  |  |  |
| Urban-rural status |  |  |
| Urban | 466,852 | 40.1% |
| Rural | 128,851 | 35.4% |

**Note: For* 162 (0.02%) patients who were retreated after initial treatment among all 672,745 patients who were treated, they are only counted as treated once.

Supplementary Figure Legends

Supplementary Figure 1a HCV Treatment Rates Among HCV RNA-Positive Patients by Age, 2014-2021

Supplementary Figure 1b. HCV Treatment Rates by FIB-4 score, 2014-2021

Supplementary Figure 1c. HIV Co-infection Status, 2014-2021

Supplementary Figure 2. Percentage of the HCV RNA-Positive Patients Treated in 2021

Receipt of treatment was determined based on a viral load decline of at least 1.2 × log10 units since the first positive HCV RNA test, indicating that treatment was initiated in the immediate period prior to the decline.
