# Supplementary material for: HCV Testing and Treatment of Adults in the United States: 2014 Through 2021—Data From Two National Commercial Testing Laboratories
Source: J Viral Hepat. 2025 Sep 29;32(11):e70087. doi: 10.1111/jvh.70087 (PMC12477661; doi:10.1111/jvh.70087)
Supplement: Supplementary file 2 — Figure S1: (a) HCV treatment rates among HCV RNA‐positive patients by age, 2014–2021. (b) HCV treatment rates by FIB‐4 score, 2014–2021. (c) HIV co‐infection status, 2014–2021. Figure S2: Percentage of the HCV RNA‐positive patients treated in 2021 receipt of treatment was determined based on a viral load decline of at least 1.2 × log10 units since the first positive HCV RNA test, indicating that treatment was initiated in the immediate period prior to the decline. [file JVH-32-0-s001.pptx]

## Slide 1
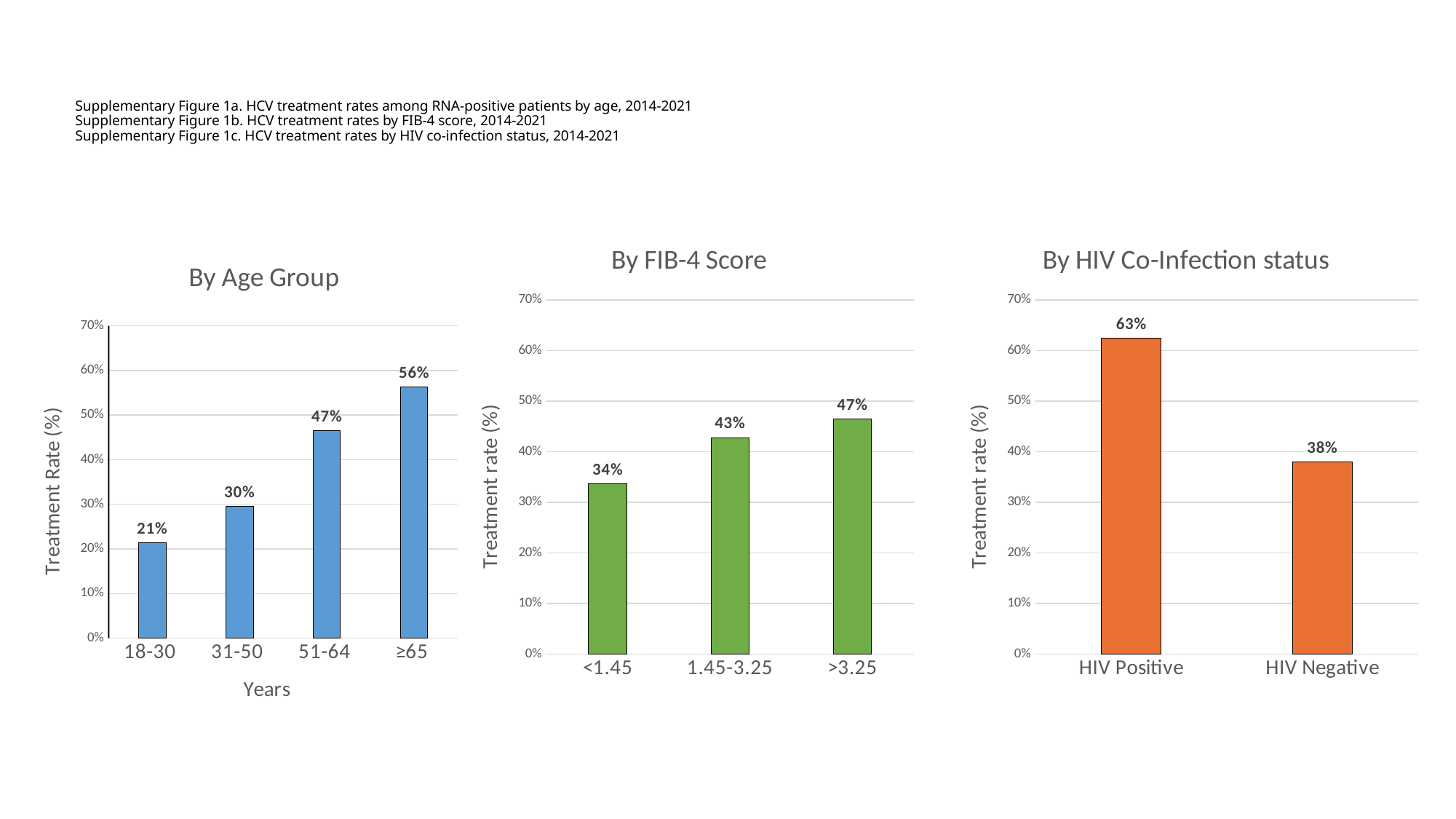

# Supplementary Figure 1a. HCV treatment rates among RNA-positive patients by age, 2014-2021 Supplementary Figure 1b. HCV treatment rates by FIB-4 score, 2014-2021 Supplementary Figure 1c. HCV treatment rates by HIV co-infection status, 2014-2021
### Chart: By FIB-4 Score
| Category | |
|---|---|
| <1.45 | 0.336 |
| 1.45-3.25 | 0.428 |
| >3.25 | 0.465 |
### Chart: By HIV Co-Infection status
| Category | |
|---|---|
| HIV Positive | 0.625 |
| HIV Negative | 0.38 |
### Chart: By Age Group
| Category | |
|---|---|
| 18-30  | 0.21309371518177692 |
| 31-50  | 0.2954184657283035 |
| 51-64  | 0.46526287091775237 |
| ≥65  | 0.5629460772341176 |

## Slide 2
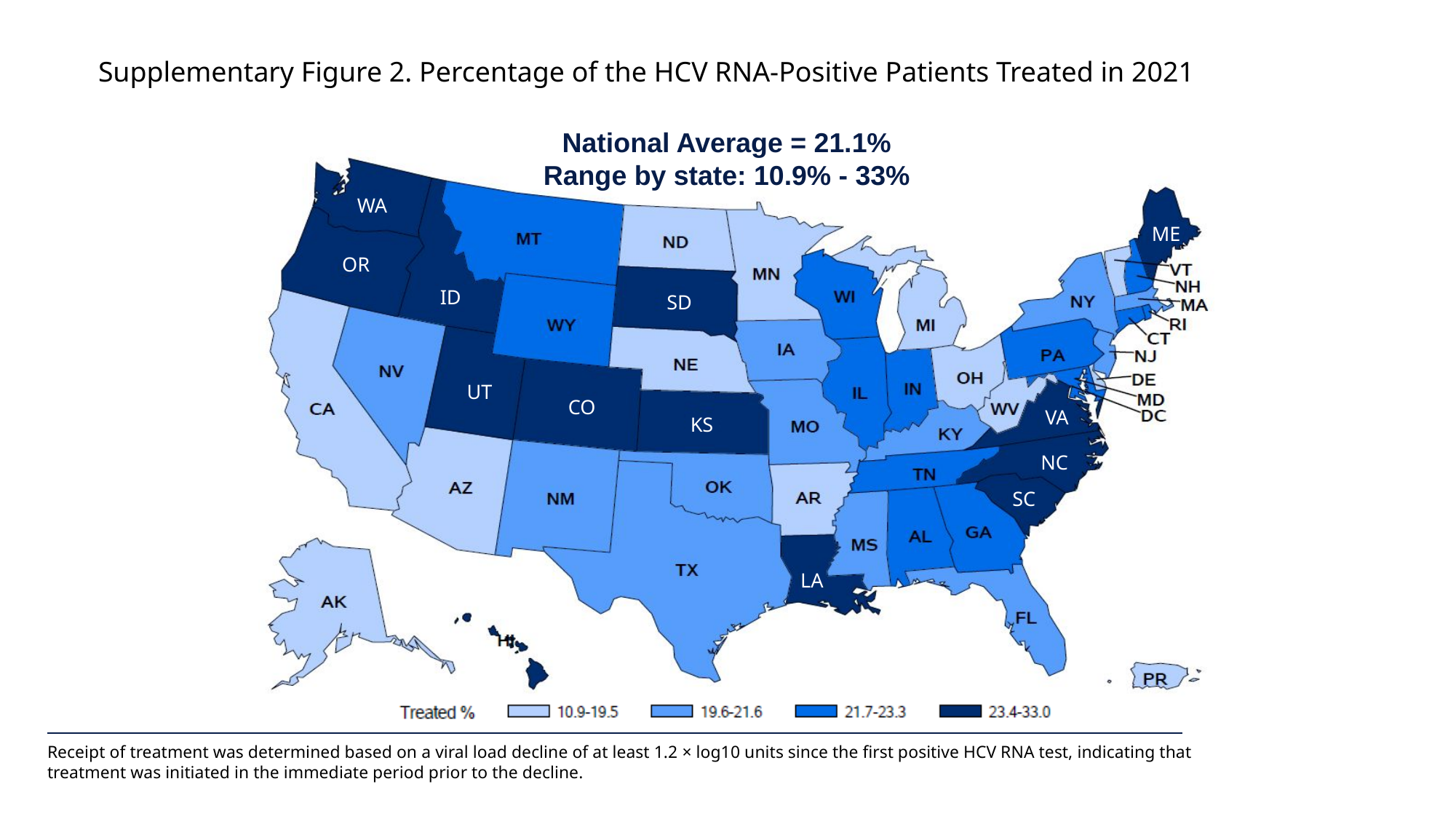

# Supplementary Figure 2. Percentage of the HCV RNA-Positive Patients Treated in 2021
National Average = 21.1%
Range by state: 10.9% - 33%
WA
ME
OR
ID
SD
UT
CO
VA
KS
NC
SC
LA
Receipt of treatment was determined based on a viral load decline of at least 1.2 × log10 units since the first positive HCV RNA test, indicating that treatment was initiated in the immediate period prior to the decline.
